# Take Pax6 for a Cortical 6-Pack

**DOI:** 10.1371/journal.pbio.1002218

**Published:** 2015-08-07

**Authors:** Roland G. Roberts

**Affiliations:** Public Library of Science, Cambridge, United Kingdom

## Abstract

This synopsis examines the implications of a new study that imposes primate-like expression of the transcription factor Pax6 on the developing mouse cortex and finds evidence of primate-like proliferation of neuronal progenitors.

Many of the higher cognitive functions of the mammalian brain depend on the activity of the neocortex—a thin and highly structured layer of cells that covers most of the outside of our brains like an intelligent helmet. Not all creatures are equally endowed, however, and comparison of the brains of different mammals shows that although the thickness of the neocortex varies relatively little between species (from 0.5 to 3 mm), more thinking power can be crammed into the same brain by making the cortex buckle and fold during development to form a convoluted sheet with a greatly increased higher surface area. The degree of folding—known as the gyration index—varies substantially between mammalian species.

Recent studies show that the Jurassic great-granddaddy of all living mammals probably had a folded brain, and that the evolutionary transition between folded and smooth neocortex has taken place on several different occasions across the mammalian family tree. Indeed, a study published last year by Wieland Huttner’s group in *PLOS Biology* showed that regardless of the size of the animal, the occurrence of folding depends on whether its brain has more than or less than a threshold value of one billion cortical neurons [1]. Under that, you stay relatively smooth; over it, you fold extensively. The gyration index seemed to track certain aspects of lifestyle; broadly speaking, mammals with smooth cortices (such as mice) tend to have smaller social groups and narrower habitat ranges, while those with convoluted cortices (such as monkeys) are party animals that can adapt to more varied habitats.

But the existence of the billion-neuron threshold begs the question as to how the number of neurons in the developing neocortex—and therefore the decision whether to fold or not to fold—is determined. Indeed, that same study went on to show that the explanation lay in the proliferative behavior of the cells that give rise to most of the neurons and glia in the adult cortex—the basal progenitors (BPs).

The cortex arises from cells that initially sit in the ventricular zone—the layer that surrounds the ventricles or chambers of the developing brain. These give rise to apical radial glia (aRGs)—cells with long processes that stretch from the inside to the outside of the developing cortex, providing a radially arranged scaffolding for neurons to follow, and dividing to generate both neurons and BPs. The BPs detach themselves and migrate up out of the ventricular zone into the overlying subventricular zone (SVZ), and this is where the interesting differences occur.

BPs take one of two forms—either basal radial glia (bRGs—long cells that resemble their apical forebears) or basal intermediate progenitors (bIPs). Moreover, they can divide in one of two ways—either “neurogenically” (giving rise to two neurons, period) or “proliferatively” (giving rise to two BPs, which are free to proliferate further). In smooth-brained mammals, neurogenic division predominates, rapidly terminating the cell lineages in a pair of neurons. In folded-brain mammals, by contrast, proliferative division predominates, resulting in extra generations of BPs and a potentially exponential increase in the eventual number of neurons. This more proliferative behavior also involves the creation of a new layer—the outer SVZ, in which the extra proliferation can take place.

This cellular explanation, however, in turn demands a molecular one, and this is where a new study from Fong Kuan Wong, Ji-Feng Fei, Wieland Huttner and colleagues—also published in *PLOS Biology*—comes in [2]. These authors were intrigued by differences in the expression of the transcription factor Pax6, which is initially needed for cell division of rodent radial glia, but is subsequently down-regulated; in primates, however, Pax6 expression is sustained, especially in the proliferative bRGs and bIPs. Thus Pax6, with an expression pattern that tightly reflects the proliferative differences that underlie folded versus smooth brains, seemed an ideal candidate.

To check this, the authors used an elegant procedure to manipulate the expression of Pax6 in the developing mouse brain. They introduced a plasmid containing an untranslatable version of the *Pax6* gene into the ventricles of mice as they developed in the uterus, driving it into the adjacent ventricular zone cells by applying an electric field (“electroporation”). The mice had been engineered to make CreER—a DNA recombinase enzyme—specifically in cells that give rise to BPs; when treated with the drug tamoxifen, the CreER recombinase would render the *Pax6* gene translatable, allowing the authors to turn on the supply of exogenous Pax6 in the BPs of the developing cortex.

The authors checked that their strategy had the desired effects, finding that exogenous Pax6 was expressed in tamoxifen-treated electroporated mice specifically in the apical progenitor cells (such as aRGs) and their progeny. Added to the endogenous Pax6, this gave a total amount of Pax6 that is three times that found in normal mice in ventricular zone apical cells. In their progeny—the BP cells in which endogenous Pax6 is normally down-regulated—the continued expression of exogenous Pax6 increased levels up to six times the normal amount.

This higher and more sustained expression pattern of Pax6 is indeed very primate-like in appearance—exactly what the researchers were hoping to achieve. To make sure that nothing had gone awry, they checked that Pax6 up-regulation didn’t grossly affect cell migration during the first 24 h of expression and that it didn’t result in widespread cell death (as has been previously reported for less stringent Pax6 overexpression).

So what was the effect of imposing primate-like Pax6 expression patterns on the developing rodent cortex? Examining the developing brains 24 h after the Pax6 boost, the authors found that more BPs in the SVZ were actively proliferating. They also found that more bRGs (rather than bIPs) were being produced, recognisable by their long processes, sometimes stretching all the way out to the pia—the membrane that covers the outer layer of the cortex.

Looking another 24 h later, the number of cells that had re-entered the cell cycle had also doubled in brains with higher Pax6 (both bRGs and bIPs), and these had a higher proliferative capacity. The number of cells that divided with a horizontal cleavage plane (a mode of division thought to favor bRG progeny) was also doubled, and there was evidence of increased numbers of bIPs. Looking even later, the offspring of the high-Pax6 cells were clearly more numerous, mostly being immature neurons in the intermediate zone.

The authors were also able to confirm many of these observations in a mouse where the tamoxifen-induced CreER recombinase in the apical progenitor cells render *Pax6* gene translatable in the mouse genome (as opposed to one introduced via a plasmid into the ventricle). This should give a more global and long-lasting effect. These mice also had an increased number of neurons, with greater proliferation in the SVZ and the overlying intermediate zone. Interestingly, this is strongly reminiscent of the extra layer (the outer SVZ) found between the SVZ and the intermediate zone in the developing cortex of mammals like primates and ferrets that have folded brains.

Therefore the take-home of the study is that when the expression of Pax6 is changed from rodent-like to primate-like, the crucial progenitor cells in the developing mouse cortex also adopt a whole suite of properties that are characteristic of the primate brain—more horizontal cleavage, more bRGs, more proliferation, and more neurons (Fig 1).

**Fig 1:**
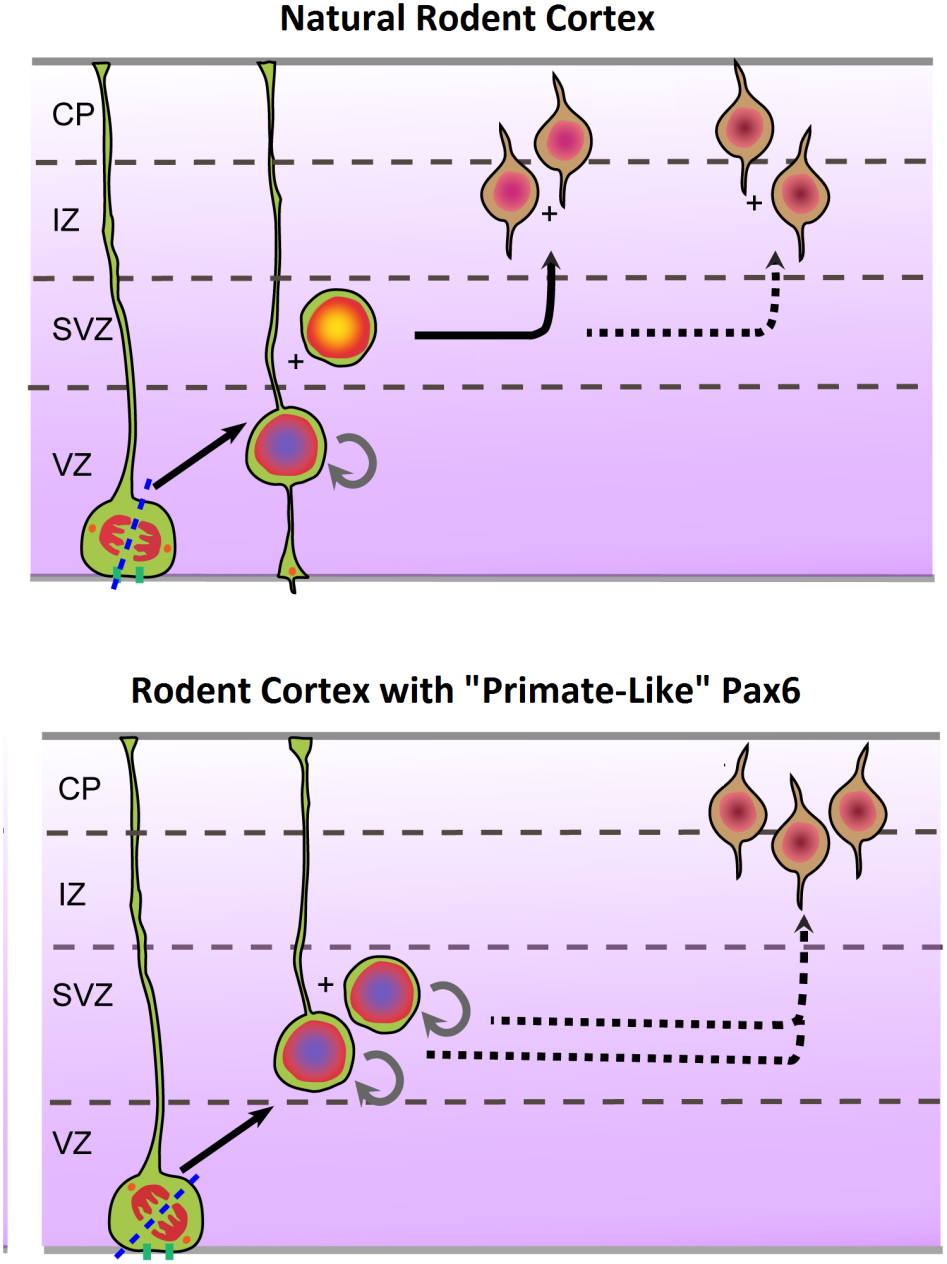
The effects of a “primate-like” Pax6 expression pattern on the mouse cortex. Each panel shows a slice through the developing mouse cortex, with the outer surface (pia) at the top and the ventricle at the bottom (VZ—ventricular zone; SVZ—subventricular zone; IZ—intermediate zone; CP—cortical plate). The upper panel shows the normal mouse pattern of development, while the lower panel shows the effects of sustaining Pax6 in a primate-like way. The effects include (from left) rotation of the aRG cleavage plane, higher numbers of bRGs, increased proliferation in the SVZ, and more neurons (modified from [2]).

These results take us considerably further than previous attempts to manipulate Pax6 levels, which have lacked the temporal and spatial specificity needed and have consequently given conflicting answers. However, unlike two other genes that have been previously shown to cause cortical expansion (Trnp1 and ARHGAP11B [3,4]), Pax6 does not cause folding in mouse. This is presumably because other factors, such as even greater proliferation in the outer layers, are needed. And although the current authors show that mice lacking Pax6 show the opposite effect—a reduction in the proportion of cortical bRGs—the crucial (and challenging) experiment would be to impose mouse-like Pax6 expression on a macaque or ferret and see if their brain shows some rodent attributes. A further question is whether the differences in Pax6 expression are intrinsic to the gene itself or are determined by species-specific differences in the genes that regulate it.

The answer is inevitably that alterations in the sequence, expression, and structure of multiple genes collaborate to achieve the delicate adjustments made by evolution on the road from folded brain to smooth or vice versa. But the authors make a convincing case for *Pax6* being one of those collaborators.

## Abbreviations

aRGs: apical radial glia
bIPs: basal intermediate progenitors
BPs: basal progenitors
bRGs: basal radial glia
SVZ: subventricular zone
